# Marked Changes in Gut Microbiota in Cardio-Surgical Intensive Care Patients: A Longitudinal Cohort Study

**DOI:** 10.3389/fcimb.2019.00467

**Published:** 2020-01-15

**Authors:** Heleen Aardema, Paola Lisotto, Alexander Kurilshikov, Janneke R. J. Diepeveen, Alex W. Friedrich, Bhanu Sinha, Anne Marie G. A. de Smet, Hermie J. M. Harmsen

**Affiliations:** ^1^Department of Critical Care, University of Groningen, University Medical Center Groningen, Groningen, Netherlands; ^2^Department of Medical Microbiology, University of Groningen, University Medical Center Groningen, Groningen, Netherlands; ^3^Department of Genetics, University of Groningen, University Medical Center Groningen, Groningen, Netherlands

**Keywords:** gut microbiota, intestinal microbiota, 16S rRNA gene sequencing, intensive care unit, critically ill, longitudinal study

## Abstract

**Background:** Virtually no studies on the dynamics of the intestinal microbiota in patients admitted to the intensive care unit (ICU) are published, despite the increasingly recognized important role of microbiota on human physiology. Critical care patients undergo treatments that are known to influence the microbiota. However, dynamics and extent of such changes are not yet fully understood. To address this topic, we analyzed the microbiota before, during and after planned major cardio surgery that, for the first time, allowed us to follow the microbial dynamics of critical care patients. In this prospective, observational, longitudinal, single center study, we analyzed the fecal microbiota using 16S rRNA gene sequencing.

**Results:** Samples of 97 patients admitted between April 2015 and November 2016 were included. In 32 patients, data of all three time points (before, during and after admission) were available for analysis. We found a large intra-individual variation in composition of gut microbiota. During admission, a significant change in microbial composition occurred in most patients, with a significant increase in pathobionts combined with a decrease in strictly anaerobic gut bacteria, typically beneficial for health. A lower bacterial diversity during admission was associated with longer hospitalization. In most patients analyzed at all three time points, the change in microbiota during hospital stay reverted to the original composition post-discharge.

**Conclusions:** Our study shows that, even with a short ICU stay, patients present a significant change in microbial composition shortly after admission. The unique longitudinal setup of this study displayed a restoration of the microbiota in most patients to baseline composition post-discharge, which demonstrated its great restorative capacity. A relative decrease in benign or even beneficial bacteria and increase of pathobionts shifts the microbial balance in the gut, which could have clinical relevance. In future studies, the microbiota of ICU patients should be considered a good target for optimisation.

## Introduction

Infections are a major threat to critical care patients leading to increased morbidity and mortality and require high antibiotic consumption (Vincent et al., 2009). Although the merits of antibiotics in this vulnerable population are undisputed, the impact of antibiotic use on the host's intestinal flora is an important concern (Wischmeyer et al., 2016). Selective digestive tract decontamination (SDD) is widely used as an infection prevention measure in Intensive Care Units (ICUs) in the Netherlands, leading to a mortality reduction compared to standard care (de Smet et al., 2009). Benefits notwithstanding (de Smet et al., 2009), SDD impacts the gut microbiota, by suppressing Gram-negative potential pathogens, *Staphylococcus aureus* (*S. aureus*) and yeasts, while maintaining anaerobic populations through selective use of antibiotics, with unclear clinical consequences (Benus et al., 2010). Besides antibiotics, several other factors inherent to ICU stay are associated with a disturbance in the host's microbiota, also called “dysbiosis,” where the balance between potentially pathogenic and beneficial bacteria is aberrant (Kitsios et al., 2017). These factors include a change in nutrition, non-antibiotic pharmacological interventions (e.g., proton pump inhibitors and vasopressors), and various invasive procedures including endotracheal intubation and surgery (Krezalek et al., 2016; Kitsios et al., 2017). Beneficial roles of the healthy gut microbiota on human physiology are complex and include nutrient metabolism, modulation of host immune responses (Sekirov et al., 2010) and protection against potential pathogens by competition (Harris et al., 2017). In a state of dysbiosis, overgrowth of potential pathogens occurs which can lead to inflammation and infection (Sekirov et al., 2010). Current culture-independent techniques allow for an in-depth analysis of the composition of gut microbiota (Sekirov et al., 2010; Harris et al., 2017).

Despite increased recognition of the important role of intestinal microbiota on health and disease (Harris et al., 2017), few studies have been published on the dynamics of gut microbiota in ICU patients using 16S rRNA gene sequencing (Iapichino et al., 2008; Zaborin et al., 2014; McDonald et al., 2016; Ojima et al., 2016; Yeh et al., 2016; Buelow et al., 2017; Lankelma et al., 2017), generally involving acute admissions for organ failure caused by infections, emergency surgery or trauma. Most of these studies (Zaborin et al., 2014; McDonald et al., 2016; Ojima et al., 2016; Yeh et al., 2016; Buelow et al., 2017; Lankelma et al., 2017) utilize a case-control design and compare the gut microbiota composition of ICU patients and healthy controls. Small pilot studies (Iapichino et al., 2008; Zaborin et al., 2014; Ojima et al., 2016; Buelow et al., 2017) as well as larger studies (McDonald et al., 2016; Yeh et al., 2016; Lankelma et al., 2017) suggest the evidence of rapid disruption of gut microbiota during ICU stay associated with a loss of diversity and overgrowth of potentially pathogenic micro-organisms, referred to here as pathobionts (Iapichino et al., 2008). Previously published data show that antibiotic-associated disturbance of microbiota can take months to restore (Lankelma et al., 2015).

The purpose of this study was to evaluate the dynamics of the composition of the gut microbiota in patients scheduled for cardiac surgery before, during and after hospital admission. Using 16S rRNA gene sequence analysis, we also evaluated a correlation between composition of intestinal microbiota and both baseline characteristics and clinical outcomes. We observed that, despite a large inter-individual variability in microbial composition at baseline, the composition of gut microbiota during hospital stay showed a concordant pattern toward decreasing microbial alpha diversity, with a relative preponderance of potentially pathogenic species. This shift was followed by full or partial post-discharge restoration. These findings are in line with available literature (Dethlefsen et al., 2008; Iapichino et al., 2008; Sekirov et al., 2010; Zaborin et al., 2014).

## Materials and Methods

### Study Population and Study Design

In this study, one hundred patients undergoing planned cardiac surgery in the University Medical Center Groningen were recruited between April 2015 and November 2016. Adult patients, scheduled for cardiac surgery involving coronary artery bypass graft (CABG) and/or valve surgery, screened in our outpatient clinic preoperatively and admitted to the ICU after surgery, were eligible for inclusion. Patient characteristics and clinical data were recorded, including occurrence of complications during ICU and hospital stay. Duration of hospital stay included stay at peripheral hospitals for further recovery. Antibiotic consumption from 3 months prior to inclusion until 8 weeks after admission was documented. All patients received peri-operative prophylaxis for 24 h with cefazolin or clindamycin. On indication (expected length of stay (LOS) in ICU ≥ 72 h and/or expected duration of mechanical ventilation for ≥ 48 h), SDD was prescribed, consisting of cefotaxime 1 gram intravenously four times daily for 4 days and a topical application of tobramycin, colistin and amphotericin B into the oropharynx and stomach throughout ICU stay as described elsewhere (de Smet et al., 2009). Patients colonized with *Staphylococcus aureus* received intranasal mupirocin ointment pre-operatively according to national protocol. We defined the following as serious adverse outcomes: ICU- and in hospital-mortality, increased (≥4 days) ICU length of stay (LOS), prolonged (≥2 days) duration of mechanical ventilation, and occurrence of bacteraemia and post-operative wound infections, including mediastinitis. If a patient had one or more of these adverse outcomes, we defined this patient to have a combined adverse outcome (CAO). Fecal samples were obtained by a research nurse at ideally three time points: pre-admission on the day of pre-operative screening (baseline or Time point 1, T1), once during admission in the ICU or ward around day 4 (Time point 2, T2), and post-admission at a post-discharge routine visit or home visit (Time point 3, T3). All available samples were included for analysis. All samples were stored directly at 4°C and transferred to −20°C within 24 h; later, samples were stored at−80°C until further processing. All procedures performed in studies involving human participants were in accordance with the ethical standards of the institutional and/or national research committee and with the 1964 Helsinki declaration and its later amendments or comparable ethical standards. Ethical approval by the Medical Ethics Committee of our institution was received (approval number METc2014/206). Informed consent was obtained from all individual participants included in the study.

### Microbiota Analysis; 16S rRNA Gene Sequencing

#### DNA Extraction and MiSeq Preparation

From the stool sample, 0.25 g was used to extract the total DNA as previously described (de Goffau et al., 2013). Subsequently, the amplification of the V3–V4 region of the 16S rRNA gene was performed using modified 341F and 806R primers (Supplementary Material 1) containing a 6-nucleotide barcode and flow-cell adaptor on the 806R primer as described elsewhere (Bartram et al., 2011). A 2 × 300 cartridge (Illumina, Eindhoven, the Netherlands) was used to perform both MiSeq library preparation and sequencing. Supplementary Material 2 represents a detailed description of the PCR protocol, DNA cleanup and the library preparation.

#### Analysis of Sequence Reads

The paired-end sequencing data received from Illumina software were processed by the software PANDAseq (version 2.5) (Masella et al., 2012) and QIIME (version 1.7.0) (Caporaso et al., 2010). Readouts with a quality score below 0.9 were discarded by PANDAseq to increase the quality of the sequence readouts. De novo OTU-picking was performed without chimera filtering with Greengenes (version 13.5) as reference database and ARB software (version 5.5) using a SILVA database (version 123) was used to check for contamination by the “kitome” on OTU level, only negligible numbers were detected (Ludwig et al., 2004; de Goffau et al., 2018).

### Statistical Analysis

The calculation of alpha diversity (Shannon index) and beta diversity was performed using R package “vegan” (version 2.5-4) on the taxonomic level of genera. The associations of microbial beta diversity vs. baseline and outcome characteristics were calculated using *adonis* (permutational multivariate analysis of variance using distance matrices), stratifying permutations for multi-time points when comparing patient characteristics, or stratifying by patients when associating beta diversity to time.

The statistical analysis of associations of baseline and outcome patient characteristics vs. alpha diversity and microbial taxa were performed using linear regression (“base” package for R) and mixed models (package “lmerTest” v. 3.1-0), when including time point or patient as random effects. The correction for false discovery rate was applied considering the number of microbial taxa included into analysis at the level of 5%, and was calculated using the Benjamini-Hochberg method.

For each layer of microbiota (alpha diversity, beta diversity, bacterial taxa), the association analysis of microbiome features with baseline and clinical characteristics was performed on three levels. On level one, we associated the microbial composition against each of baseline and outcome characteristics adjusting for age and sex. For alpha diversity and bacterial taxa, a linear mixed-model approach was used with sampling time (before, during or after admission, encoded as 3-level factor) as random effect. For bacterial beta diversity, we stratified permutations by time group using “*strata”* parameter for *adonis* function (R package “*vegan” v*. 2.5–4). On level two, we re-evaluated the associations between microbiota features with clinical outcomes (CAO, LOS at the hospital, antibiotic post discharge and therapeutic antibiotics), additionally adjusting for all the baseline characteristics (age, gender, co-morbidities, BMI, antibiotic pre-admission). On level three, we associated the outcome measures with microbiota composition in each time point separately.

The graphs were made with R package ‘ggplot2’ (version 3.1.1).

## Results

**Figure 1:**
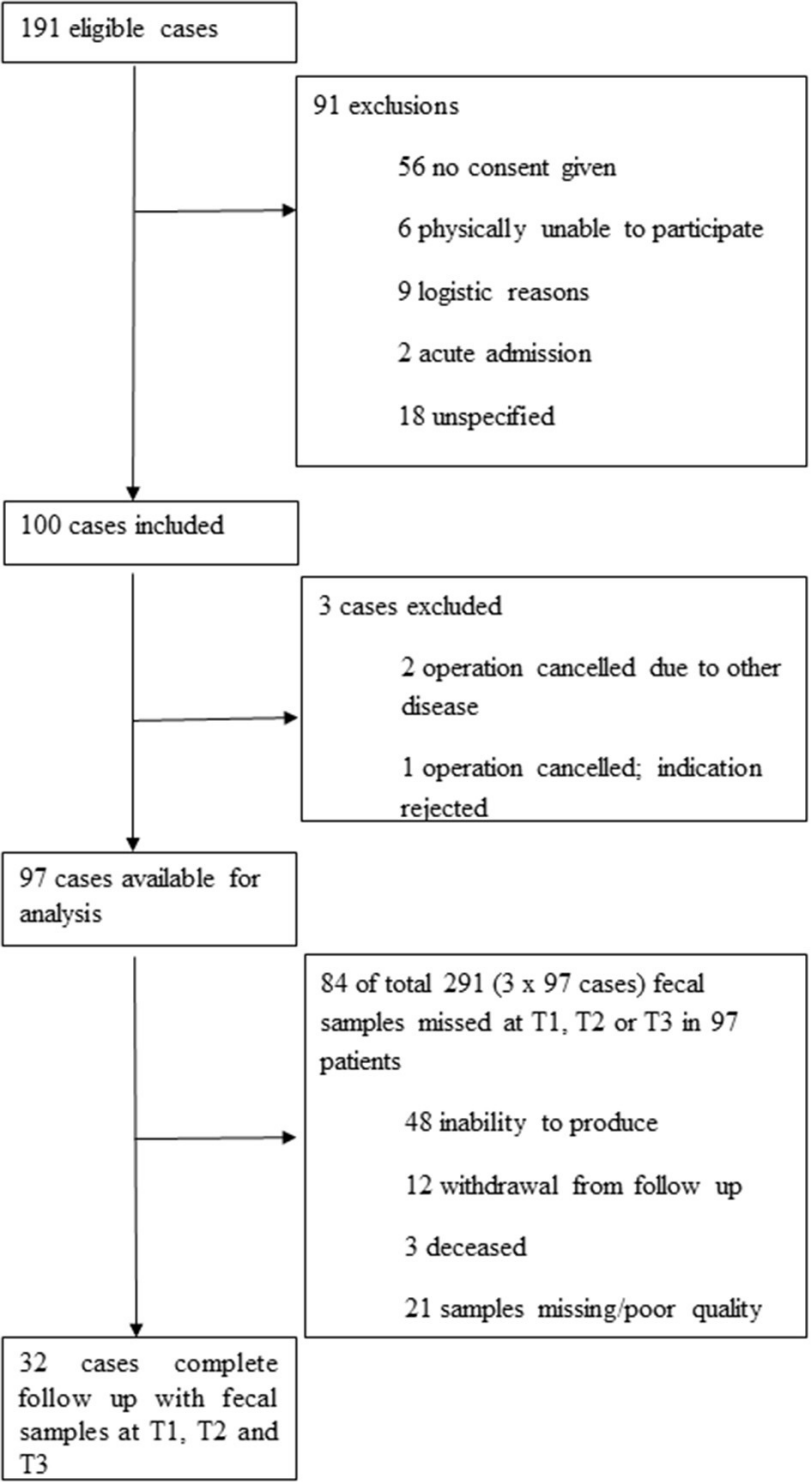
Patient and sample flowchart.

Inclusion of patients and collection of samples are summarized in Figure 1. Detailed baseline characteristics and outcome parameters are summarized in Table 1. Ninety-seven patients were included. Pre-admission fecal samples were obtained from 92 patients, during admission at a median of 4 days post-surgery (interquartile range (IQR), 3–5 days) from 53 patients; and post-admission at a median of 71 days post-surgery (IQR, 57–88 days) from 83 patients. From 32 patients, fecal samples obtained at all time points were available for analysis. Reasons for missing samples are shown in Figure 1. No patient withdrew consent. Details on antibiotic consumption are listed in Supplementary Material 3.

**Table 1:** Baseline characteristics and outcome.

**Variable**	***N* = 97**
Age, years	68 [62–73]
Sex (male)	73 (75%)
BMI, kg/m^2^	27.5 ± 4.6
Planned operation	
Coronary Artery Bypass Graft (CABG), N (%)	39 (40.2%)
CABG + Valve repair, N (%)	17 (17.5%)
Valve repair, N (%)	41 (42.3%)
Co-morbidity	
Yes, N (%)	35 (36.1%)
Pulmonary disease	10 (10.3%)
Diabetes mellitus	22 (22.7%)
Chronic kidney failure	2 (2.1%)
Solid malignancy	4 (4.1%)
Hemato-oncologic malignancy	0 (0%)
Immunosuppression	2 (2.1%)
Alcohol/substance abuse	1 (1.0%)
Antibiotics in 3 months pre-admission	
Yes	12 (12.4%)
Unknown	8 (8.2%)
No	77 (79.4%)
Euroscore	1.77 [1.07–2.91]
APACHE IV	48 [39–57]
Exposure to antibiotics during admission	
24 h of cefazolin^a^	96 (99.0%)
SDD^b^	7 (7.2%)
≥ 1 day antibiotics	14 (14.4%)
Length of stay (LOS) Intensive Care Unit (ICU), days	1 [1–2]
LOS hospital, days^c^	9 [8–13]
Need of vasopressors > 1 day	7 (7.2%)
Duration of mechanical ventilation	
< 24 h (hrs)	92 (94.8%)
24–48 h	2 (2.1%)
2–5 days	1 (1.0%)
> 5 days	2 (2.1%)
Bacteraemia	3 (3.1%)
Peri-operative wound Infection	3 (3.1%)
Re-exploration	7 (7.2%)
Re-admission ICU	9 (9.3%)
Died during admission	1 (1.0%)
Combined adverse outcome^d^	13 (13.4%)
Antibiotics in 3 months post-discharge	
Yes	22 (22.7%)
Unknown	2 (2.1%)
No	73 (75.3%)

*Data presented as median with [IQR], numbers with (%), or mean ± standard deviation, as appropriate*.

a *One additional patient received peri-procedural clindamycin due to penicillin allergy*.

b *SDD denotes Selective Decontamination of the Digestive tract*.

c *Available in 94 patients*.

d *Combined adverse outcome defined as one or more of the following: ICU mortality, in-hospital mortality, length of stay in ICU ≥ 4 days, duration of mechanical ventilation ≥ 2 days, occurrence of Post-Operative Wound Infection (POWI), including mediastinitis, or bacteraemia*.

### Lower Alpha Diversity Associated With Longer Hospital Stay and Antibiotic Use After Discharge

**Figure 2:**
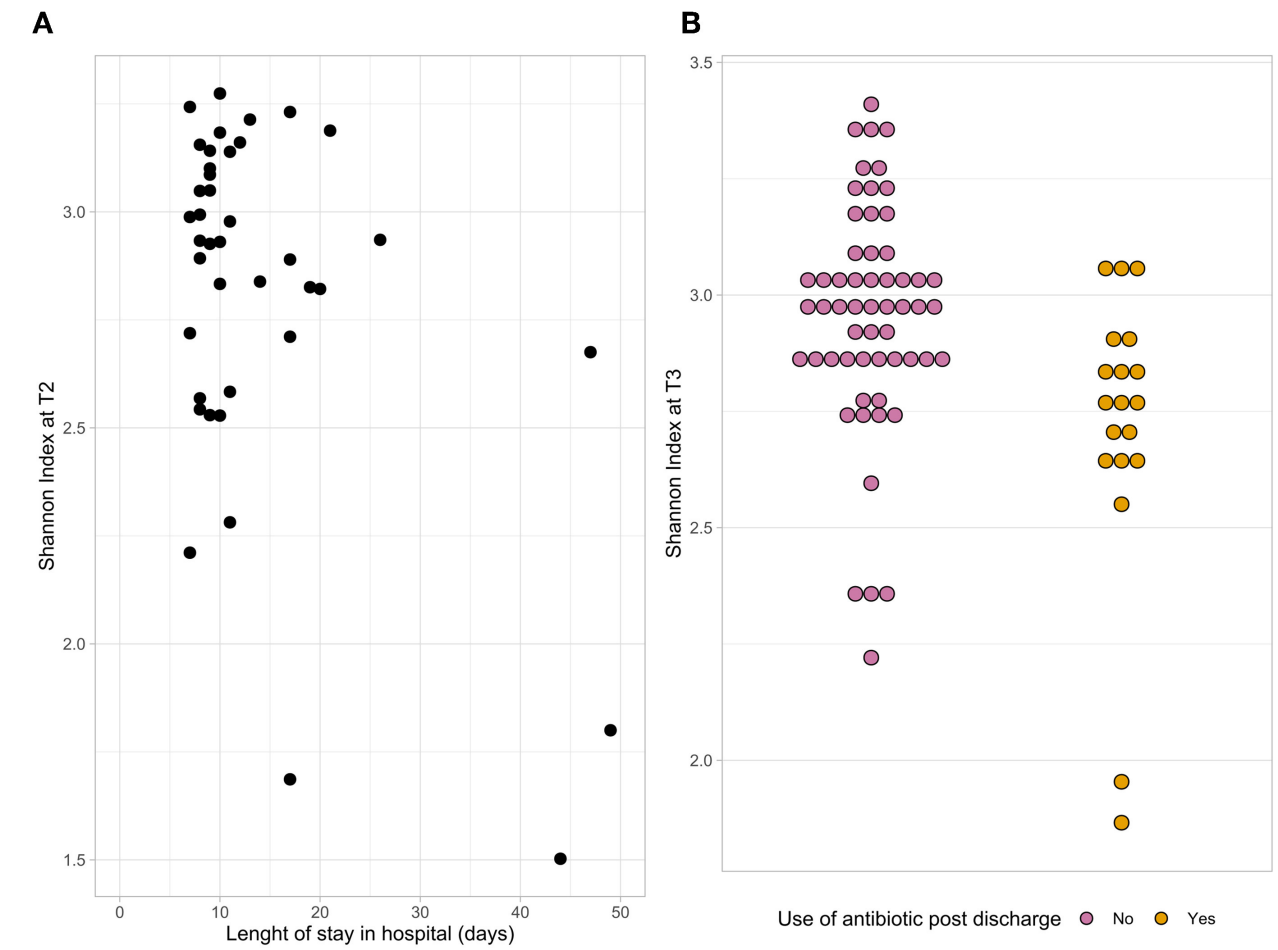
The dot plots represent the association between the Shannon index at T2 and length of hospital stay **(A)** and the association between the Shannon index at T3 and antibiotic use after discharge **(B)**.

The microbial composition of fecal samples was determined using 16S rRNA gene analysis. Bacterial diversity, measured as Shannon index, significantly changed at T2 compared to baseline (*p* = 0.02). A linear mixed-effect model was used to determine associations between alpha diversity and patient baseline and outcome characteristics. First, we tested each variable separately. Length of hospital stay, use of SDD and use of antibiotics post-discharge were found to be significantly associated with the Shannon index (*p* < 0.05). When all these variables were combined together in a single model, only length of hospital stay and post-discharge antibiotic use remained significant; therefore, it was tested which time point they were associated to. A lower alpha diversity at T2 was associated with a longer hospital stay (Figure 2A) and at T3 with antibiotic use after discharge (Figure 2B).

### The Microbiota Composition Shifts During Admission but Reverts Back After Discharge

**Figure 3:**
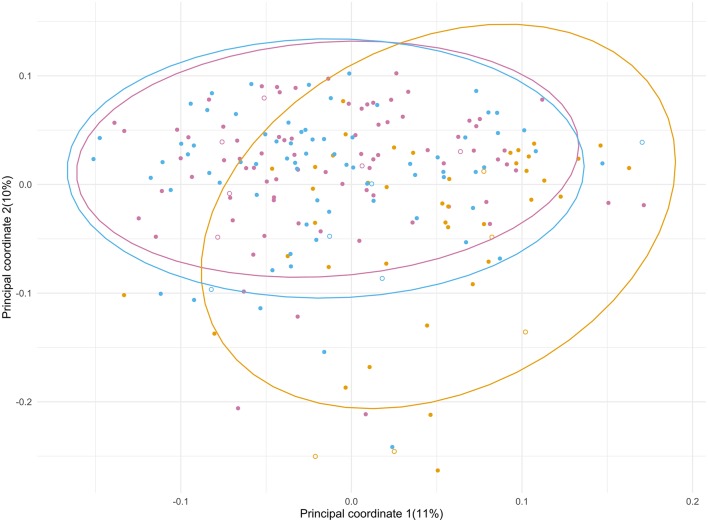
Principal coordinate analysis of the microbial composition of the samples from ICU patients significantly changed over the course of the study period (*p* < 0.001). The different time points are depicted in purple (T1), yellow (T2) and blue (T3). Patients who did (7) and did not (90) receive SDD treatment are indicated with open and closed symbols, respectively.

Principal Coordinate Analysis (PCoA) (Figure 3) was performed to identify a clustering pattern of the microbial compositions; the baseline (T1) and post-admission (T3) samples were spread over a large area in the PCoA plot, shifting from those taken days after surgery (T2) with an overall significant change in composition over time (*p* < 0.001). During admission, a shift in composition of gut microbiota from baseline was seen (*p* < 0.001), with an increase in principal component 1 and a decrease in principal component 2, away from the original profile. Plotted samples after discharge (T3) showed a difference with T2 (*p* < 0.001). At T3 the samples seem to return to the baseline situation in the PCoA plot indicating recovery of the microbial composition, although statistically, there is still a significant difference (*p* = 0.021). In order to determine how much of the variance observed among patients could be explained by the available baseline characteristics and outcomes, the PERMANOVA analysis (Supplementary Material 4) was performed. We looked into each time point to investigate how the relative contribution of these characteristics would change during the study period. We found that treatment with SDD could explain almost 7% of the variation exhibited during admission (T2), while the combined adverse outcome alone was accountable for almost 5% at T2. The use of antibiotics during admission and post-discharge, collectively, would explain 6% of variation of the microbial composition after discharge (T3).

### Associations Between Bacterial Taxa and Patient Characteristics

Next, we performed the association study between bacterial genera with baseline characteristics and outcome parameters, results can be found in detail in Supplementary Material 5. When estimating each of the baseline and outcome characteristics separately (level 1), we observed associations of several bacterial taxa with: (i) BMI, (ii) comorbidities (immunosuppression, solid malignancy and chronic kidney failure), (iii) therapeutic antibiotic use during admission and (iv) hospital length of stay. When adjusting for baseline characteristics (level 2), we observed associations with: (i) SDD, (ii) the use of antibiotics during admission and (iii) hospital length of stay.

### Bacterial Dynamics Over Time

**Figure 4:**
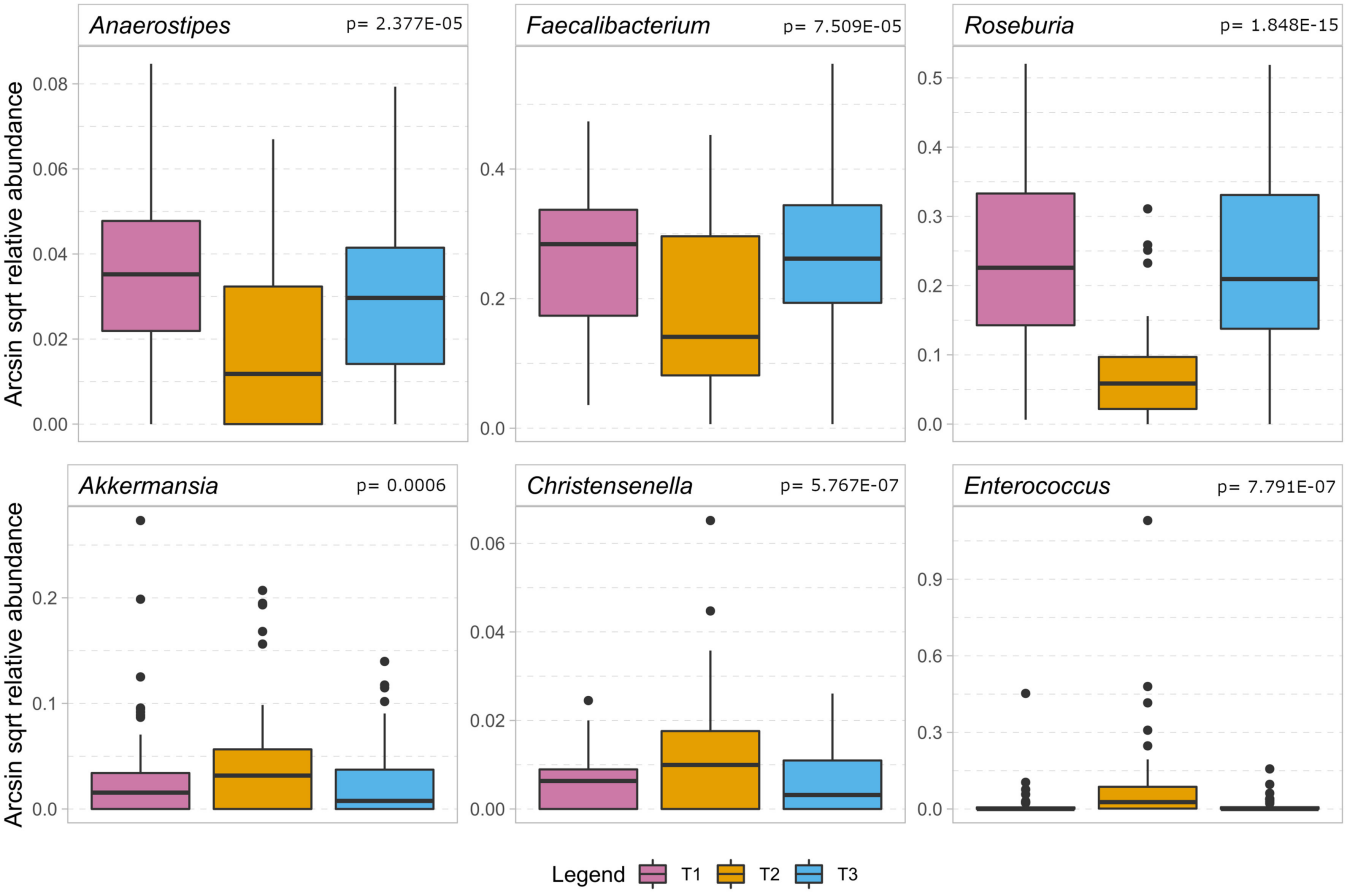
The boxplot shows the results of the association analysis of the gut microbiota in ICU patients over time. In the top row a selection of beneficial butyrate-producing gut bacteria (for details, cf. Supplementary Material 6) show a significant decrease during the stay at the intensive care unit. In the bottom row we show bacteria that significantly increase during hospitalization of which *Enterococcus* is considered a pathobiont while the other two are regarded as beneficial to gut health. In the analysis, only taxa remaining after application of FDR < 0.05 are considered significant and the *p*-value of the quadratic component of the model is depicted in the headings.

Figure 4 indicates that during the treatment timeline, strictly anaerobic short-chain fatty acid-producing gut bacteria typically beneficial for health, such as *Faecalibacterium, Anaerostipes, Blautia*, and *Roseburia* (De Vos and de Vos, 2012; Rajilic-Stojanovic and de Vos, 2014; Reichardt et al., 2018), decreased significantly at T2. In contrast, pathobionts or members of oral microbiota such as *Enterococcus, Eggerthella, Peptococcus*, and *Rothia* (Weber and Gold, 2003; Zaura et al., 2009; Yeh et al., 2016; Si et al., 2017; Ugarte-Torres et al., 2018), showed a substantial increase at T2. Interestingly, the tendency to increase in T2 was also observed for the obligate anaerobic gut microbes, such as *Akkermansia, Bifidobacterium*, and *Methanobrevibacter*, which are known to be the most stable in gut microbiota under external perturbations and assumed dependent on host genetic makeup (Goodrich et al., 2016) (Supplementary Material 6). Further, we specifically looked at the dynamics of *Enterobacteriaceae* in the SDD sub cohort. We found no consistent change over time, however, at T2 the relative contribution was lower than 1% (data not shown) in this group of patients.

### Clinical Course and Gut Microbiota Composition of Patients With the Lowest Shannon Index

**Figure 5:**
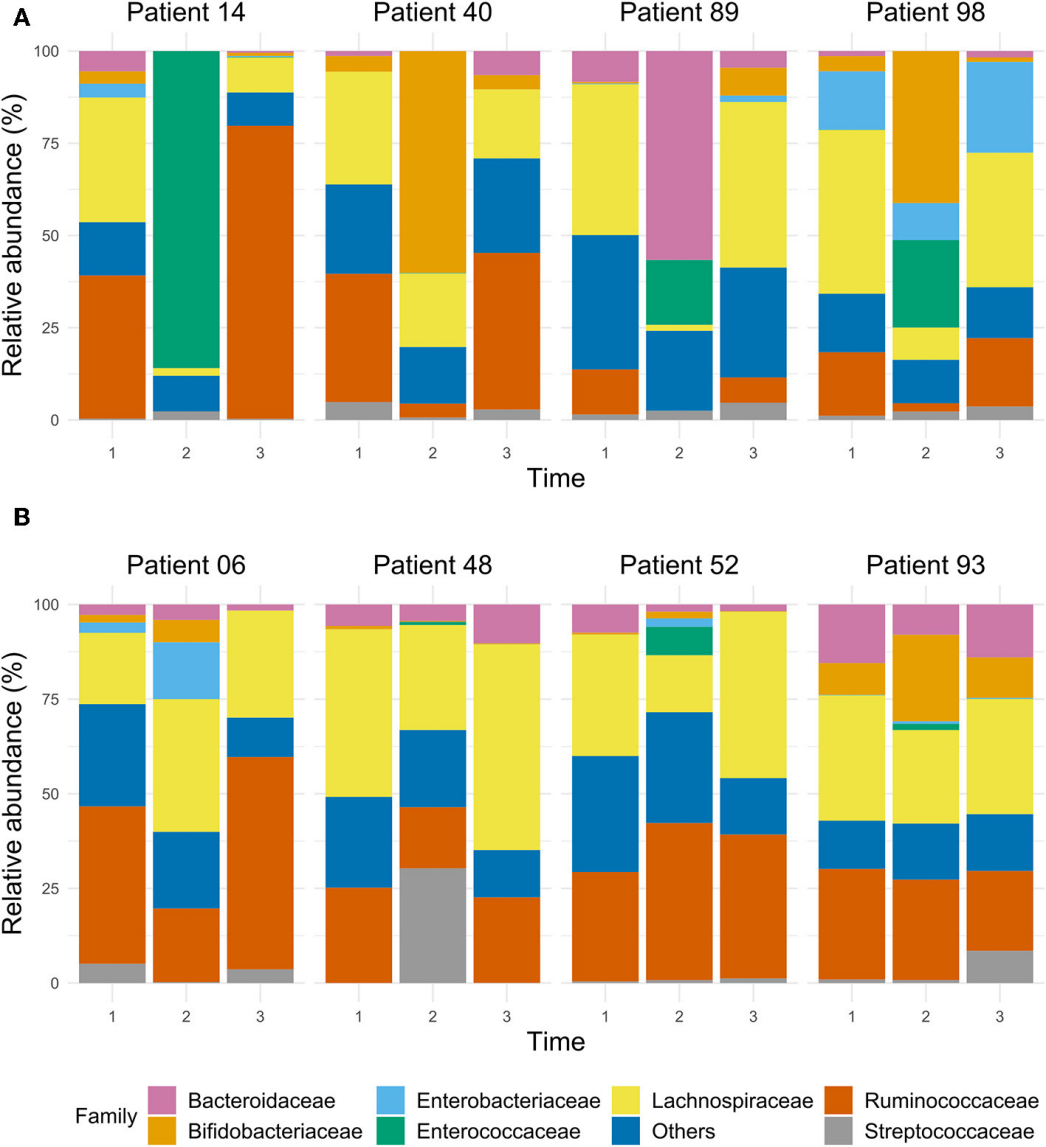
The bar chart of selected samples represents the relative abundance of the main taxa, expressed at family level, in the gut microbiota of ICU patients whose microbiota dramatically decreased according to their Shannon index during the stay at the ICU **(A)**, and of ICU patients who had a stable Shannon index over time **(B)**.

The dynamics of the gut microbiota composition in four patients (#14, #40, #89, #98) who had the lowest Shannon index at T2 is depicted in Figure 5. Patient #14 had a complicated course after CABG with sternal dehiscence, mediastinitis, sternal closure with pectoralis major flaps, complicated by a wound infection, with cultures yielding *Enterobacter cloacae, S. aureus* and *Enterococcus faecium*, myocardial ischemia with decompensated heart failure and *S. aureus* bacteraemia, needing prolonged treatment with several classes of antibiotics (Supplementary Material 3), also including treatment with SDD, a long hospital stay and readmission. In this patient, the relative abundance of *Enterococcaceae* was 82% at T2. In patient #40, who had an uneventful clinical course, 60% of the gut microbiota was dominated by *Bifidobacteriaceae* at T2, while at T1 and T3, *Bifidobacteriaceae*'s contribution to the total flora was 4%. Patient #89, who had a prolonged ICU stay due to shock caused by heart failure and subsequent renal failure, showed a relatively high abundance of *Bacteroidaceae* (57%) at T2, while this was 8 and 5% at T1 and T3, respectively. Likewise, there was a relatively high abundance of *Enterococcaceae* (18%) at T2; at T1 and T3 no *Enterococcaceae* were detected. Patient #98, with an uneventful course, showed a remarkable relative abundance of *Enterobacteriaceae* (16%) at T1, with a decrease to 10% at T2, but an increase to 25% at T3, while at T2, a relative abundance of *Enterococcaceae* (24%) was detected; this was 0% at T1 and T3.

## Discussion

Only few studies on the dynamics of intestinal microbiota in critical care patients using culture-independent methods have been published (Iapichino et al., 2008; Zaborin et al., 2014; McDonald et al., 2016; Ojima et al., 2016; Yeh et al., 2016; Buelow et al., 2017; Lankelma et al., 2017), despite evolving interest in microbiota. Results provide a promising insight in potential diagnostic and therapeutic possibilities for the prevention or cure of infections (Harris et al., 2017; Kitsios et al., 2017). This is the first prospective, observational longitudinal study analyzing the dynamics of microbiota in patients receiving major elective extra-intestinal surgery who post-operatively were admitted to the ICU.

In order to analyse bacterial diversity, we included the Shannon index. As previously reported and in line with other studies (Lankelma et al., 2017), we found a significantly lower Shannon index during than before admission. In our longitudinal setup, we found the Shannon index after discharge not to be significantly different from before admission. The main differences were observed between the first two time points, confirming that all interventions related to hospital admission for elective surgery have a major impact on a patient's microbiota. The length of stay at the hospital was associated with a lower Shannon index, especially in the microbiota sampled at T2, suggesting the potential use of the gut microbiota data for an intervention that might prevent post-surgery complications. A lower Shannon index at T3 was associated with use of antibiotics post-discharge indicating the strong effect that this therapy has on the microbial gut diversity.

The gut microbial composition of patients before, during and after admission showed a large intra-individual variability. These changes occurred not only in patients with a complicated course, but also in those with an uneventful post-operative recovery. Relatively few patients (13 individuals, i.e., 13.4%) had a predefined CAO. The median ICU stay was only 1 day, rendering this cohort to be different from the previous studies on dynamics of microbiota in ICU patients involving mainly acute admissions due to sepsis, surgery and trauma (Iapichino et al., 2008; Zaborin et al., 2014; McDonald et al., 2016; Ojima et al., 2016; Yeh et al., 2016; Buelow et al., 2017; Lankelma et al., 2017). During hospital stay, we observed a consistent pattern of changes in microbiota, characterized by: (i) a substantial drop in abundance of many bacteria from the Lachnospiraceae family, including the genera *Blautia, Roseburia* and *Dorea*; (ii) an increase in potential pathobionts, including enterococci; (iii) a relative increase in abundance of non-pathogenic gut commensal genera known to be stable over time, such as mucin-degrading *Akkermansia, Bifidobacterium*, and the archeon *Methanobrevibacter*. Many factors potentially responsible for variations in the microbiota composition may contribute to the observed dynamic pattern (Zhernakova et al., 2016; Kitsios et al., 2017). All patients were exposed to multiple interventions including major surgery, dietary changes and antibiotic use for at least a short period. Some patients had more protracted courses with a longer ICU stay with use of SDD as an infection prevention measure, longer courses of antibiotics, and re-interventions. Although SDD is partially aimed at *Enterobacteriaceae*, no significant change in their abundance was seen in this small sub cohort likely due to sample size and possibly due to the low relative contribution of *Enterobacteriaceae* to the gut microbiota composition. The most pronounced dynamic change of the gut microbiota during hospital stay in our cohort is a decrease in abundance of bacterial members of healthy microbiota, mostly butyrate producers. Taking into account their roles as energy providers for colonocytes, together with their known anti-inflammatory properties (Lankelma et al., 2015), we postulate that this decrease in butyrate producers may have a direct impact on clinical outcome. These results confirm and complement previously published studies in disease- and treatment-associated microbiota dynamics in different patient cohorts (Iapichino et al., 2008; Zaborin et al., 2014; McDonald et al., 2016; Ojima et al., 2016; Yeh et al., 2016; Buelow et al., 2017; Lankelma et al., 2017).

In most patients analyzed at all time points, the change in microbiota during hospital stay reverted to the original composition post-discharge, showing the flexibility of the gut microbiota and its great restorative capacity. We observed the associations between microbial dynamics and clinical metadata but they appear not to be clinically relevant. Our data thus do not allow to state whether the microbial composition at baseline could influence outcome and whether a state of dysbiosis is a cause or an effect of critical illness. Dysbiosis was previously found to be associated with an increased risk for infection and adverse outcome. For instance, in a cohort of 301 medical ICU patients, disruption of microbiota with *Enterococcus* domination (≥30% relative abundance) as assessed by 16S rRNA gene sequencing at ICU admission was associated with a 22% increased risk for all-cause infection or death (Freedberg et al., 2018). Additionally, dysbiosis is associated with chronic disease states such as inflammatory bowel disease, obesity, diabetes and cardiovascular disease (Wischmeyer et al., 2016). In case of elective surgery, influencing or preventing a state of dysbiosis before surgery might lead to a better outcome. The use of novel probiotic strains to boost anaerobic butyrate-producing bacteria could improve resilience to the effect of surgery and hospital stay in general on the microbiota, or even simple nutritional advice or supplementation with vitamins could improve the condition of the patients' microbiota. A possible role for fecal microbiota transplantation (FMT) also merits consideration (McClave et al., 2018); from a recent study, autologous FMT showed a more rapid recovery than probiotics (Suez et al., 2018). Therefore, “profiling” the microbiota of a patient before admission could be of relevance. On the other hand, our data showed that microbiota profiling during admission is much more promising from a clinical perspective, due to its potential for predicting post-surgery clinical outcomes, such as length of hospital stay.

The strength of this study is its longitudinal design: ideally, patients were sampled three times, thereby serving as their own control. This study has several limitations. We had a relatively large number of missing fecal samples. Although in the context of mentioned previously published studies our cohort was relatively large, the sample size is still to be considered small. In future studies, as noted by others (Lankelma et al., 2017), the use of rectal swabs for sequencing could be an attractive alternative especially in large-scale studies, with results similar to those from stool samples (Budding et al., 2014). Differentiating the individual factors responsible for changes in intestinal microbiota is not possible due to the omnipresent use of antibiotics. Also, no (control) patient is withheld from surgery. We did not assess the contribution of all factors potentially influencing the intestinal microbiota composition of the patient, e.g., the use of proton pump inhibitors was not evaluated (Kitsios et al., 2017). Moreover, disentangling the many factors contributing to the disruption of the intestinal microbiota composition is limited by the inherent combination of some of these factors (Dickson, 2016); a patient with a prolonged course due to infectious complications with hemodynamic instability will likely be treated with prolonged antibiotics, possibly from different antibiotic classes, for instance. How these individual factors contributed to the observed association between length of hospital stay and lower alpha diversity at T2, is therefore impossible to say in this study design. Antibiotic use during stay in peripheral hospitals to which patients with uncomplicated courses were transferred was not documented. The use of 16S rRNA gene sequencing limits the resolution of microbial data to the level of genera and, for some taxonomic groups, species, and does not allow profiling bacterial strains or identifying abundances of metabolic functions. Viruses and fungi, although of presumed clinical relevance, were also not targeted by this method. As this study was carried out in a single center setting involving cardiac surgery patients, our results might not be generalisable to all critical care patients. However, despite these limitations, our project is a step forward in understanding the dynamics of the microbiota and holds promising views on potential diagnostic and therapeutic options for ICU patients.

Our study shows that gut microbiota composition of elective surgery patients admitted to the ICU undergoes significant changes. The decrease in beneficial bacteria and the relative increase of specific pathobionts shift the microbial balance to dysbiosis in the gut, which has clinical relevance. Therefore, optimizing the microbiota of patients admitted to the ICU should be considered in future studies.

## Data Availability Statement

The datasets used and/or analyzed during the current study are registered within the Sequence Read Archive under the BioProject number PRJNA578267 and are thus freely accessible.

## Ethics Statement

The studies involving human participants were reviewed and approved by Medical Ethics Committee, University of Groningen, University Medical Center Groningen, approval number METc2014/206. The patients/participants provided their written informed consent to participate in this study.

## Author Contributions

HA, AF, BS, AS, and HH designed the study. HA supervised patient recruitment and patient sample flow. PL and JD performed 16S rRNA gene sequencing and analyzed these data. HH supervised sequencing and data processing. AK, PL, and HA performed data analysis. AF, BS, AS, and HH supervised data analysis. HA and PL wrote the first draft of the manuscript. AF, BS, JD, AS, HH, and AK provided input to the draft and approved the final version of the manuscript.

### Conflict of Interest

The authors declare that the research was conducted in the absence of any commercial or financial relationships that could be construed as a potential conflict of interest.
