# Supplementary material for: Marked Changes in Gut Microbiota in Cardio-Surgical Intensive Care Patients: A Longitudinal Cohort Study
Source: Front Cell Infect Microbiol. 2020 Jan 15;9:467. doi: 10.3389/fcimb.2019.00467 (PMC6974539; doi:10.3389/fcimb.2019.00467)
Supplement: Supplementary file 1 [file Data_Sheet_1.PDF]

Supplementary material 1

V3_F_modified	aatgatacggcgaccaccgagatct	acactctttccctacacgacgctcttccgatct		NNNNCCTACGGGAGGCAGCAG
V4_1R	caagcagaagacggcatacgagat	**ATCACG**	gtgactggagttcagacgtgtgctcttccgatct	GGACTACHVGGGTWTCTAAT
V4_2R	caagcagaagacggcatacgagat	**CGATGT**	gtgactggagttcagacgtgtgctcttccgatct	GGACTACHVGGGTWTCTAAT
V4_3R	caagcagaagacggcatacgagat	**TTAGGC**	gtgactggagttcagacgtgtgctcttccgatct	GGACTACHVGGGTWTCTAAT
V4_4R	caagcagaagacggcatacgagat	**TGACCA**	gtgactggagttcagacgtgtgctcttccgatct	GGACTACHVGGGTWTCTAAT
V4_5R	caagcagaagacggcatacgagat	**ACAGTG**	gtgactggagttcagacgtgtgctcttccgatct	GGACTACHVGGGTWTCTAAT
V4_6R	caagcagaagacggcatacgagat	**GCCAAT**	gtgactggagttcagacgtgtgctcttccgatct	GGACTACHVGGGTWTCTAAT
V4_7R	caagcagaagacggcatacgagat	**CAGATC**	gtgactggagttcagacgtgtgctcttccgatct	GGACTACHVGGGTWTCTAAT
V4_8R	caagcagaagacggcatacgagat	**ACTTGA**	gtgactggagttcagacgtgtgctcttccgatct	GGACTACHVGGGTWTCTAAT
V4_9R	caagcagaagacggcatacgagat	**GATCAG**	gtgactggagttcagacgtgtgctcttccgatct	GGACTACHVGGGTWTCTAAT
V4_10R	caagcagaagacggcatacgagat	**TAGCTT**	gtgactggagttcagacgtgtgctcttccgatct	GGACTACHVGGGTWTCTAAT
V4_11R	caagcagaagacggcatacgagat	**GGCTAC**	gtgactggagttcagacgtgtgctcttccgatct	GGACTACHVGGGTWTCTAAT
V4_12R	caagcagaagacggcatacgagat	**CTTGTA**	gtgactggagttcagacgtgtgctcttccgatct	GGACTACHVGGGTWTCTAAT
V4_13R	caagcagaagacggcatacgagat	**AGTACG**	gtgactggagttcagacgtgtgctcttccgatct	GGACTACHVGGGTWTCTAAT
V4_14R	caagcagaagacggcatacgagat	**TCAGTC**	gtgactggagttcagacgtgtgctcttccgatct	GGACTACHVGGGTWTCTAAT
V4_15R	caagcagaagacggcatacgagat	**TTGAGC**	gtgactggagttcagacgtgtgctcttccgatct	GGACTACHVGGGTWTCTAAT
V4_16R	caagcagaagacggcatacgagat	**AAGCGA**	gtgactggagttcagacgtgtgctcttccgatct	GGACTACHVGGGTWTCTAAT
V4_17R	caagcagaagacggcatacgagat	**TCCTCA**	gtgactggagttcagacgtgtgctcttccgatct	GGACTACHVGGGTWTCTAAT
V4_18R	caagcagaagacggcatacgagat	**GGTTGT**	gtgactggagttcagacgtgtgctcttccgatct	GGACTACHVGGGTWTCTAAT
V4_19R	caagcagaagacggcatacgagat	**TGAGGT**	gtgactggagttcagacgtgtgctcttccgatct	GGACTACHVGGGTWTCTAAT
V4_20R	caagcagaagacggcatacgagat	**TACCGT**	gtgactggagttcagacgtgtgctcttccgatct	GGACTACHVGGGTWTCTAAT
V4_21R	caagcagaagacggcatacgagat	**CCAACT**	gtgactggagttcagacgtgtgctcttccgatct	GGACTACHVGGGTWTCTAAT
V4_22R	caagcagaagacggcatacgagat	**AGAGAG**	gtgactggagttcagacgtgtgctcttccgatct	GGACTACHVGGGTWTCTAAT
V4_23R	caagcagaagacggcatacgagat	**CACTTG**	gtgactggagttcagacgtgtgctcttccgatct	GGACTACHVGGGTWTCTAAT
V4_24R	caagcagaagacggcatacgagat	**TCAAGG**	gtgactggagttcagacgtgtgctcttccgatct	GGACTACHVGGGTWTCTAAT
V4_25R	caagcagaagacggcatacgagat	**AGTGGT**	gtgactggagttcagacgtgtgctcttccgatct	GGACTACHVGGGTWTCTAAT
V4_26R	caagcagaagacggcatacgagat	**GACACT**	gtgactggagttcagacgtgtgctcttccgatct	GGACTACHVGGGTWTCTAAT
V4_27R	caagcagaagacggcatacgagat	**CCTTCT**	gtgactggagttcagacgtgtgctcttccgatct	GGACTACHVGGGTWTCTAAT
V4_28R	caagcagaagacggcatacgagat	**GGATAA**	gtgactggagttcagacgtgtgctcttccgatct	GGACTACHVGGGTWTCTAAT
V4_29R	caagcagaagacggcatacgagat	**CCTTAA**	gtgactggagttcagacgtgtgctcttccgatct	GGACTACHVGGGTWTCTAAT
V4_30R	caagcagaagacggcatacgagat	**CAAGAA**	gtgactggagttcagacgtgtgctcttccgatct	GGACTACHVGGGTWTCTAAT
V4_31R	caagcagaagacggcatacgagat	**GTTGAA**	gtgactggagttcagacgtgtgctcttccgatct	GGACTACHVGGGTWTCTAAT
V4_32R	caagcagaagacggcatacgagat	**TCACAA**	gtgactggagttcagacgtgtgctcttccgatct	GGACTACHVGGGTWTCTAAT
V4_33R	caagcagaagacggcatacgagat	**AGTCAA**	gtgactggagttcagacgtgtgctcttccgatct	GGACTACHVGGGTWTCTAAT
V4_34R	caagcagaagacggcatacgagat	**CGAATA**	gtgactggagttcagacgtgtgctcttccgatct	GGACTACHVGGGTWTCTAAT
V4_35R	caagcagaagacggcatacgagat	**GCTATA**	gtgactggagttcagacgtgtgctcttccgatct	GGACTACHVGGGTWTCTAAT
V4_36R	caagcagaagacggcatacgagat	**GAGTTA**	gtgactggagttcagacgtgtgctcttccgatct	GGACTACHVGGGTWTCTAAT
V4_37R	caagcagaagacggcatacgagat	**TTGGTA**	gtgactggagttcagacgtgtgctcttccgatct	GGACTACHVGGGTWTCTAAT

V4_38R	caagcagaagacggcatacgagat	**AACGTA**	gtgactggagttcagacgtgtgctcttccgatct	GGACTACHVGGGTWTCTAAT
V4_39R	caagcagaagacggcatacgagat	**GTACTA**	gtgactggagttcagacgtgtgctcttccgatct	GGACTACHVGGGTWTCTAAT
V4_40R	caagcagaagacggcatacgagat	**CATCTA**	gtgactggagttcagacgtgtgctcttccgatct	GGACTACHVGGGTWTCTAAT
V4_41R	caagcagaagacggcatacgagat	**TGTAGA**	gtgactggagttcagacgtgtgctcttccgatct	GGACTACHVGGGTWTCTAAT
V4_42R	caagcagaagacggcatacgagat	**ATCAGA**	gtgactggagttcagacgtgtgctcttccgatct	GGACTACHVGGGTWTCTAAT
V4_43R	caagcagaagacggcatacgagat	**ACATGA**	gtgactggagttcagacgtgtgctcttccgatct	GGACTACHVGGGTWTCTAAT
V4_44R	caagcagaagacggcatacgagat	**TAGACA**	gtgactggagttcagacgtgtgctcttccgatct	GGACTACHVGGGTWTCTAAT
V4_45R	caagcagaagacggcatacgagat	**GAGAAT**	gtgactggagttcagacgtgtgctcttccgatct	GGACTACHVGGGTWTCTAAT
V4_46R	caagcagaagacggcatacgagat	**CTCAAT**	gtgactggagttcagacgtgtgctcttccgatct	GGACTACHVGGGTWTCTAAT
V4_47R	caagcagaagacggcatacgagat	**AGGTAT**	gtgactggagttcagacgtgtgctcttccgatct	GGACTACHVGGGTWTCTAAT
V4_48R	caagcagaagacggcatacgagat	**TTGCAT**	gtgactggagttcagacgtgtgctcttccgatct	GGACTACHVGGGTWTCTAAT
V4_49R	caagcagaagacggcatacgagat	**TGGATT**	gtgactggagttcagacgtgtgctcttccgatct	GGACTACHVGGGTWTCTAAT
V4_50R	caagcagaagacggcatacgagat	**ACCATT**	gtgactggagttcagacgtgtgctcttccgatct	GGACTACHVGGGTWTCTAAT
V4_51R	caagcagaagacggcatacgagat	**CTAGTT**	gtgactggagttcagacgtgtgctcttccgatct	GGACTACHVGGGTWTCTAAT
V4_52R	caagcagaagacggcatacgagat	**AGTGTT**	gtgactggagttcagacgtgtgctcttccgatct	GGACTACHVGGGTWTCTAAT
V4_53R	caagcagaagacggcatacgagat	**TCTCTT**	gtgactggagttcagacgtgtgctcttccgatct	GGACTACHVGGGTWTCTAAT
V4_54R	caagcagaagacggcatacgagat	**GTAAGT**	gtgactggagttcagacgtgtgctcttccgatct	GGACTACHVGGGTWTCTAAT
V4_55R	caagcagaagacggcatacgagat	**CAATGT**	gtgactggagttcagacgtgtgctcttccgatct	GGACTACHVGGGTWTCTAAT
V4_56R	caagcagaagacggcatacgagat	**ATTCGT**	gtgactggagttcagacgtgtgctcttccgatct	GGACTACHVGGGTWTCTAAT
V4_57R	caagcagaagacggcatacgagat	**ATGACT**	gtgactggagttcagacgtgtgctcttccgatct	GGACTACHVGGGTWTCTAAT
V4_58R	caagcagaagacggcatacgagat	**ACTTCT**	gtgactggagttcagacgtgtgctcttccgatct	GGACTACHVGGGTWTCTAAT
V4_59R	caagcagaagacggcatacgagat	**CATAAG**	gtgactggagttcagacgtgtgctcttccgatct	GGACTACHVGGGTWTCTAAT
V4_60R	caagcagaagacggcatacgagat	**TTCTAG**	gtgactggagttcagacgtgtgctcttccgatct	GGACTACHVGGGTWTCTAAT
V4_61R	caagcagaagacggcatacgagat	**AAGATG**	gtgactggagttcagacgtgtgctcttccgatct	GGACTACHVGGGTWTCTAAT
V4_62R	caagcagaagacggcatacgagat	**TATGTG**	gtgactggagttcagacgtgtgctcttccgatct	GGACTACHVGGGTWTCTAAT
V4_63R	caagcagaagacggcatacgagat	**AATTGG**	gtgactggagttcagacgtgtgctcttccgatct	GGACTACHVGGGTWTCTAAT
V4_64R	caagcagaagacggcatacgagat	**TAATCG**	gtgactggagttcagacgtgtgctcttccgatct	GGACTACHVGGGTWTCTAAT
V4_65R	caagcagaagacggcatacgagat	**ACTAAC**	gtgactggagttcagacgtgtgctcttccgatct	GGACTACHVGGGTWTCTAAT
V4_66R	caagcagaagacggcatacgagat	**TGTTAC**	gtgactggagttcagacgtgtgctcttccgatct	GGACTACHVGGGTWTCTAAT
V4_67R	caagcagaagacggcatacgagat	**ATACAC**	gtgactggagttcagacgtgtgctcttccgatct	GGACTACHVGGGTWTCTAAT
V4_68R	caagcagaagacggcatacgagat	**CTTATC**	gtgactggagttcagacgtgtgctcttccgatct	GGACTACHVGGGTWTCTAAT
V4_69R	caagcagaagacggcatacgagat	**AGATTC**	gtgactggagttcagacgtgtgctcttccgatct	GGACTACHVGGGTWTCTAAT
V4_70R	caagcagaagacggcatacgagat	**ACGGAA**	gtgactggagttcagacgtgtgctcttccgatct	GGACTACHVGGGTWTCTAAT
V4_71R	caagcagaagacggcatacgagat	**TGCGAA**	gtgactggagttcagacgtgtgctcttccgatct	GGACTACHVGGGTWTCTAAT
V4_72R	caagcagaagacggcatacgagat	**GACCAA**	gtgactggagttcagacgtgtgctcttccgatct	GGACTACHVGGGTWTCTAAT
V4_73R	caagcagaagacggcatacgagat	**CTGTCA**	gtgactggagttcagacgtgtgctcttccgatct	GGACTACHVGGGTWTCTAAT
V4_74R	caagcagaagacggcatacgagat	**GCAGAT**	gtgactggagttcagacgtgtgctcttccgatct	GGACTACHVGGGTWTCTAAT
V4_75R	caagcagaagacggcatacgagat	**TCGTGT**	gtgactggagttcagacgtgtgctcttccgatct	GGACTACHVGGGTWTCTAAT
V4_76R	caagcagaagacggcatacgagat	**GAACCT**	gtgactggagttcagacgtgtgctcttccgatct	GGACTACHVGGGTWTCTAAT

V4_77R	caagcagaagacggcatacgagat	**GTCATG**	gtgactggagttcagacgtgtgctcttccgatct	GGACTACHVGGGTWTCTAAT
V4_78R	caagcagaagacggcatacgagat	**GATAGC**	gtgactggagttcagacgtgtgctcttccgatct	GGACTACHVGGGTWTCTAAT
V4_79R	caagcagaagacggcatacgagat	**AAGTCC**	gtgactggagttcagacgtgtgctcttccgatct	GGACTACHVGGGTWTCTAAT
V4_80R	caagcagaagacggcatacgagat	**ATTGCC**	gtgactggagttcagacgtgtgctcttccgatct	GGACTACHVGGGTWTCTAAT
V4_81R	caagcagaagacggcatacgagat	**CCGAGA**	gtgactggagttcagacgtgtgctcttccgatct	GGACTACHVGGGTWTCTAAT
V4_82R	caagcagaagacggcatacgagat	**CGCTGA**	gtgactggagttcagacgtgtgctcttccgatct	GGACTACHVGGGTWTCTAAT
V4_83R	caagcagaagacggcatacgagat	**GGCACA**	gtgactggagttcagacgtgtgctcttccgatct	GGACTACHVGGGTWTCTAAT
V4_84R	caagcagaagacggcatacgagat	**CGTGCA**	gtgactggagttcagacgtgtgctcttccgatct	GGACTACHVGGGTWTCTAAT
V4_85R	caagcagaagacggcatacgagat	**GGCCTT**	gtgactggagttcagacgtgtgctcttccgatct	GGACTACHVGGGTWTCTAAT
V4_86R	caagcagaagacggcatacgagat	**CCTGGT**	gtgactggagttcagacgtgtgctcttccgatct	GGACTACHVGGGTWTCTAAT
V4_87R	caagcagaagacggcatacgagat	**CAGGCT**	gtgactggagttcagacgtgtgctcttccgatct	GGACTACHVGGGTWTCTAAT
V4_88R	caagcagaagacggcatacgagat	**GTCGCT**	gtgactggagttcagacgtgtgctcttccgatct	GGACTACHVGGGTWTCTAAT
V4_89R	caagcagaagacggcatacgagat	**GCGTAG**	gtgactggagttcagacgtgtgctcttccgatct	GGACTACHVGGGTWTCTAAT
V4_90R	caagcagaagacggcatacgagat	**CTGGAG**	gtgactggagttcagacgtgtgctcttccgatct	GGACTACHVGGGTWTCTAAT
V4_91R	caagcagaagacggcatacgagat	**CTACGG**	gtgactggagttcagacgtgtgctcttccgatct	GGACTACHVGGGTWTCTAAT
V4_92R	caagcagaagacggcatacgagat	**ACACCG**	gtgactggagttcagacgtgtgctcttccgatct	GGACTACHVGGGTWTCTAAT
V4_93R	caagcagaagacggcatacgagat	**GTTCCG**	gtgactggagttcagacgtgtgctcttccgatct	GGACTACHVGGGTWTCTAAT
V4_94R	caagcagaagacggcatacgagat	**CAGCAC**	gtgactggagttcagacgtgtgctcttccgatct	GGACTACHVGGGTWTCTAAT
V4_95R	caagcagaagacggcatacgagat	**CCGTTC**	gtgactggagttcagacgtgtgctcttccgatct	GGACTACHVGGGTWTCTAAT
V4_96R	caagcagaagacggcatacgagat	**GCATCC**	gtgactggagttcagacgtgtgctcttccgatct	GGACTACHVGGGTWTCTAAT
V4_97R	caagcagaagacggcatacgagat	**TACGCC**	gtgactggagttcagacgtgtgctcttccgatct	GGACTACHVGGGTWTCTAAT

Lowercase letters denote adapter sequences necessary for binding to the flow cell, underlined lowercase are binding sites for the Illumina sequencing primers, bold uppercase highlight the index sequences (all the indexes were obtained from Illumina) and regular uppercase are the V3 region forward primer 341F and the V4 region reverse primers 806R. The inclusion of four maximally degenerated bases ("NNNN") maximizes diversity during the first four bases of the run.

Supplementary material 2

Reaction conditions consisted of an initial 94 °C for 3 min followed by 32 cycles of 94 °C for 45 sec, 50 °C for 60 sec, and 72 °C for 90 sec, and a final extension of 72 °C for 10 min. An agarose gel confirmed the presence of the product (band at ~465 base pairs) in successfully amplified samples. The remainder of the PCR product (~45 µl) of each sample was mixed thoroughly with 25 µl Agencourt AMPure XP magnetic beads (Beckman Coulter) and were incubated at room temperature for 5 minutes. Beads were subsequently separated from the solution by placing the tubes in a magnetic bead separator for 2 minutes. After discarding the cleared solution the beads were washed twice by suspending them in 200 µl freshly prepared 80% ethanol, incubating the tubes for 30s in the magnetic bead separator and subsequently discarding the cleared solution. The pellet was then air dried for 15 minutes and suspended in 52.5 µl 10 mM Tris HCl pH 8.5 buffer. Fifty µl of the cleared up solution are subsequently transferred to a new tube. The DNA concentration of each sample was done using a Qubit® 2.0 fluorometer (www.invitrogen.com/qubit) and the remainder of the sample was stored at -20 °C until library normalization. Library normalization was done the day before running samples on the MiSeq by making 2 nM dilutions of each sample. Samples were pooled together by combining 5 µl of each diluted sample. Ten µl of the pooled samples and 10 µl 0.2 M NaOH were subsequently combined and incubated for 5 minutes to denature the sample DNA. To this, 980 µl of the HT1 buffer from the MiSeq 2x300 kit is was subsequently added. A denatured diluted PhiX solution was made by combining 2 µl of a 10 nM PhiX library with 3 µl 10 mM Tris HCl pH 8.5 buffer with 0.1% Tween 20. These 5 µl were mixed with 5 µl 0.2 M NaOH and incubated for 5 minutes at room temperature. These 10 µl were subsequently mixed with 990 µl HT1 buffer. From the diluted sample pool, 150 µl were combined with 50 µl of the diluted PhiX solution and further diluted by adding 800 µl HT1 buffer. Finally, 600 µl of the prepared library were loaded into the sample loading reservoir of the MiSeq 2x300 cartridge.

Supplementary material 3

Classes of antibiotics received by each patient before (a), during (b) or after (c) hospital admittance.

	Aminoglycosides	Carbapenems	Cephalosporins[2]	Clavulinic acid	Co-trimoxazole	Macrolids	Metronidazole	Penicillins	Quinolones	Tetracyclines	Tazobactam	Topical A/P/T[1]	Vancomycin	Clindamycin[3]	Linezolid	Rifampicin	Unknown[4]
#1			b														
#2			b														c
#3			b														
#4			b														
#5			b														
#6			b	b					b								
#7			b														
#8			b														
#9			b														c
#10			b														
#11			b														
#12			b					c									
#13			b														
#14		c	b					c	b			b	b c	b	b		
#15			b														
#16			b														c
#17			b														
#19			b														
#20			b														
#21			b	c													
#22			b														a
#23			b														
#24			b									b					
#25			b					c									
#26			b					a									
#27			b														
#28			b														
#29			b														
#30			b														
#31			b														
#32			b														
#33			b														
#34			b					c									a

	**Aminoglycosides**	**Carbapenems**	**Cephalosporins**[2]	**Clavulinic acid**	**Co-trimoxazole**	**Macrolids**	**Metronidazole**	**Penicillins**	**Quinolones**	**Tetracyclines**	**Tazobactam**	**Topical A/P/T**[1]	**Vanconycin**	**Clindamycin**[3]	**Linezolid**	**Rifampicin**	**Unknown**[4]
#35			**b**														
#36			**b**														
#37			**b**														
#38			**b**	**a**													
#39			**b**									**b**					
#40			**b**														
#41			**b**														
#42			**b**							**a**							
#43			**b**														
#44			**b**					**a**									
#45			**b**		**b**			**b**			**b**						**c**
#46			**b**														**a**
#47		**c**	**b**		**c**												
#48			**b**														**c**
#49			**b**	**b**				**b c**			**b**						**a**
#50			**b**														
#51			**b**														
#52			**b**							**a**							**a**
#53			**b**														**c**
#54			**b**														
#55			**b**														**c**
#56			**b**														
#57			**b**									**b**					
#58			**b**														
#59			**b**														
#60			**b**														
#61			**b**														**a c**
#62			**b**														**a c**
#63			**b**														
#64			**b**														
#65			**b**														
#66			**b**														
#67			**b**														
#68			**b**					**c**									
#69			**b**														
#70			**b**														
#71			**b**														
#72			b														

	Aminoglycosides	Carbapenems	Cephalosporins[2]	Clavulinic acid	Co-trimoxazole	Macrolids	Metronidazole	Penicillins	Quinolones	Tetracyclines	Tazobactam	Topical A/P/T[1]	Vanconycin	Clindamycin[3]	Linezolid	Rifampicin	Unknown[4]
#73			**b**					**a**									
#74			**b**														
#76														**b**			
#77			**b**														
#78			**b**														
#79			**b**														
#80			**b**														
#81			**b**														
#82			**b**														
#83			**b**				**b**	**b**			**b**		**b**				
#84			**b**														
#86			**b**									**b**					
#87	**b**		**b**					**b**	**b**				**b**	**b**		**b**	
#88			**b**														
#89			**b**									**b**					
#90			**b**														
#91			**b**					**b**			**b**	**b**	**b**				
#92			**b**														
#93			**b**														
#94			**b**														
#95			**b**														**c**
#96			**b**														
#97			**b**														
#98			**b**							**c**							
#99			**b**														
#100			**b**														

[1]Topical A/P/T denotes topical application of amphotericin B, polymyxin, tobramycin in the context of selective decontamination of the digestive tract, by protocol also including systemic application of a cephalosporin. [2]Ninety-six patients received perioperative prophylaxis (a cephalosporin); [3]one patient (#76) received clindamycin as perioperative prophylaxis. [4]"Unknown" was registered when a patient stated to have received an antibiotic (before or after hospital stay), but its name could not be retrieved.

Supplementary material 4				
**Level 1**	**Variable**	**R^2**	**P-value**	**P-value adjusted**
	BMI	0.008420848	0.03449655	0.04785885
	SDD	0.015861554	0.00089991	0.00649935
	EUROSCORE	0.005207392	0.38276172	0.41010185
	APACHE	0.002831469	0.91250875	0.91250875
	AB pre admission	0.009825415	0.02519748	0.0419958
	AB during admission	0.011442846	0.00289971	0.00869913
	AB post admission	0.010997872	0.00429957	0.01074893
	LOS	0.016584206	0.00059994	0.00649935
	CAO	0.01334495	0.00129987	0.00649935
	Pulmonary disease	0.005808058	0.22667733	0.26155077
	Diabetes mellitus	0.008349447	0.03509649	0.04785885
	Chronic kidney failure	0.009805689	0.0089991	0.01687331
	Solid malignancy	0.011317233	0.00289971	0.00869913
	Immunosoppression	0.012578389	0.0069993	0.0149985
	Alcohol/illicit drug abuse	0.007252143	0.06139386	0.07674233

Level 1: Percentage of variance explained by each variable separately

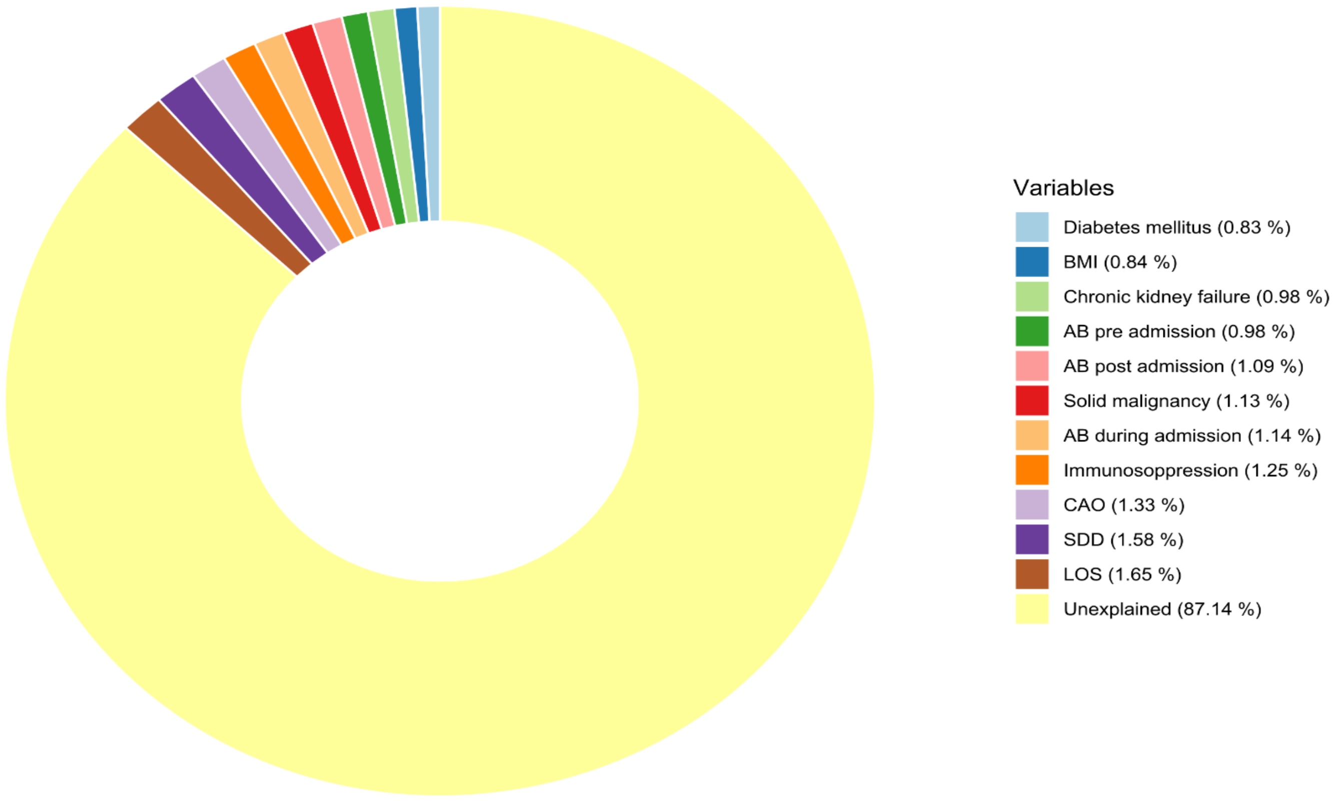

Supplementary material 4

Level 2	Variable	R^2	P-value
	BMI	0.01009687	0.01529847
	SDD	0.01591906	0.00089991
	AB during admission	0.01428485	0.00089991
	AB post admission	0.01318333	0.00149985
	LOS	0.01505776	0.0019998
	CAO	0.01180346	0.00649935

Level 2: Percentage of variance explained by all the variables together

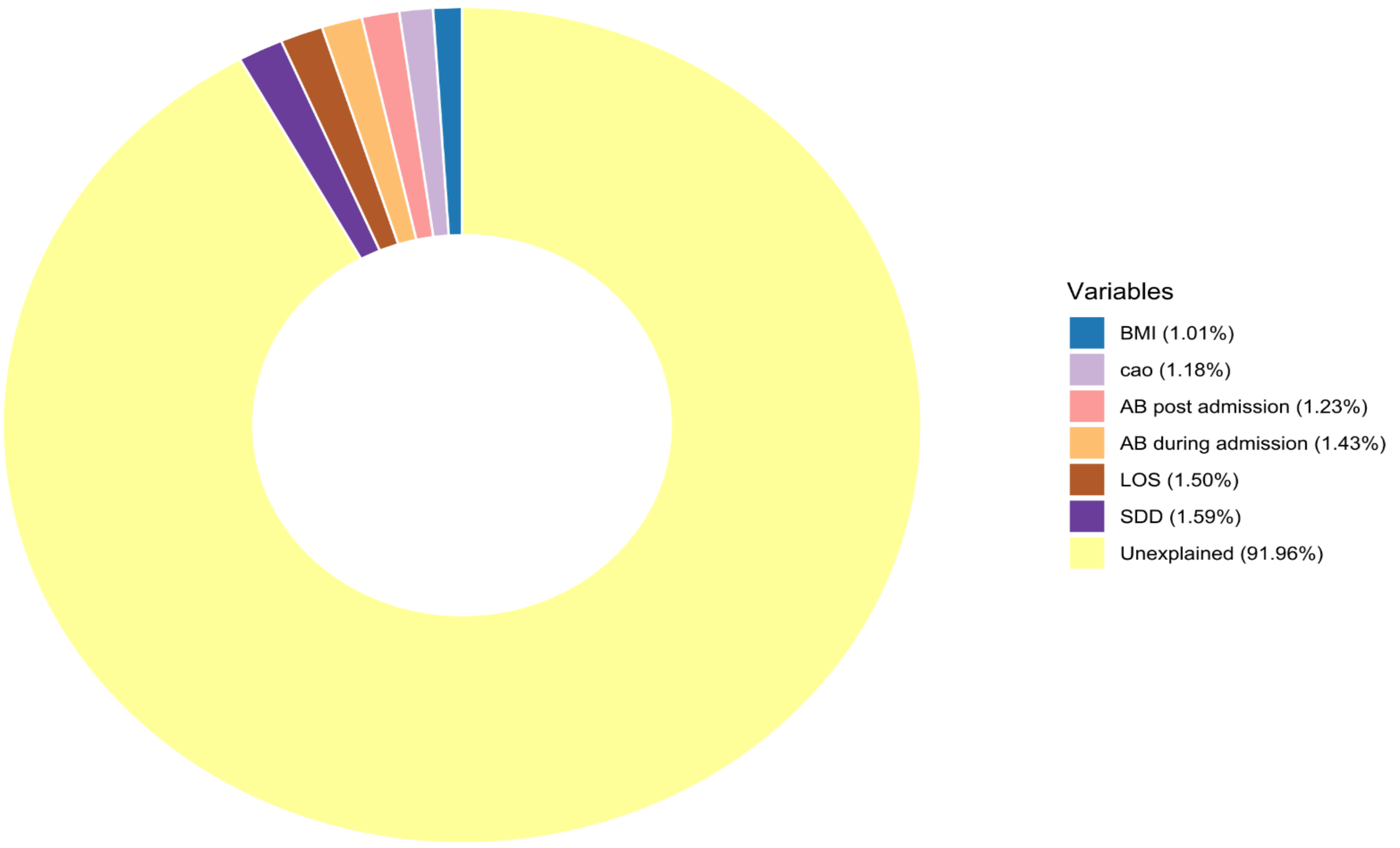

Supplementary material 4							
**Level 3**	**Variable**	**R^2_T1**	**P-value_T1**	**R^2_T2**	**P-value_T2**	**R^2_T3**	**P-value_T3**
	BMI	0.0135391	0.46453546	0.02037377	0.646353646	0.02048294	0.141858142
	SDD	0.01495817	0.33066933	0.0669292	0.005994006	0.01787005	0.234765235
	AB during admission	0.01831876	0.18681319	0.04381511	0.05994006	0.03136523	0.014985015
	AB post admission	0.01392735	0.42757243	0.0270532	0.35964036	0.03040475	0.008991009
	LOS	0.02423924	0.05094905	0.0423465	0.072927073	0.02780047	0.03996004
	CAO	0.01122007	0.63036963	0.04767171	0.045954046	0.02805348	0.017982018

Level 3: Percentage of variance explained by all the variables together at T2

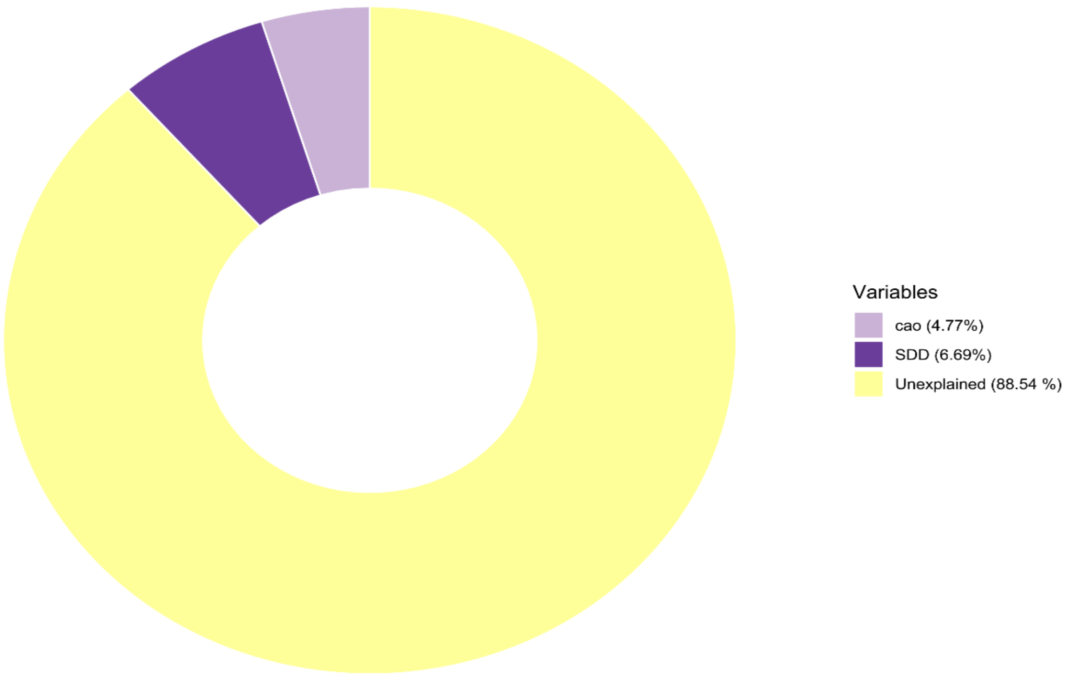

Level 3: Percentage of variance exlplained by all the variables together at T3

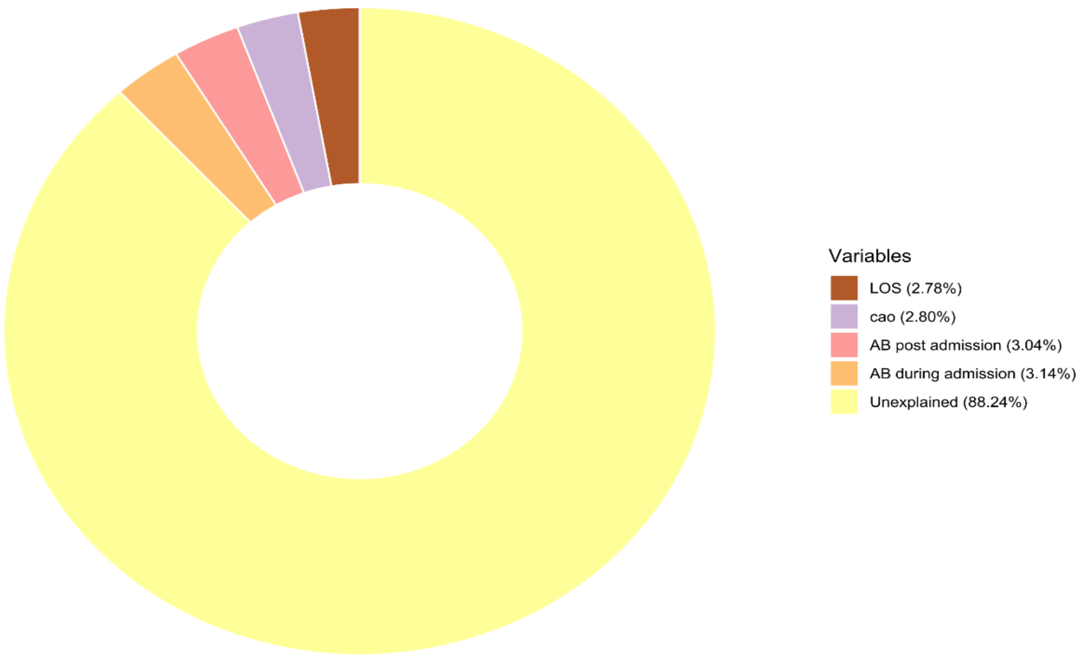

Supplementary material 5

Level 1

Taxon	Variable	Coefficient	P-value adjusted
k__Bacteria;p__Firmicutes;c__Clostridia;o__Clostridiales;f__Lachnospiraceae;g__Dorea	BMI	0.0018515	0.0450689
k__Bacteria;p__Firmicutes;c__Clostridia;o__Clostridiales;f__Veillonellaceae;g__Acidaminococcus	BMI	0.001023	0.0492613
k__Bacteria;p__Firmicutes;c__Clostridia;o__Clostridiales;f__Veillonellaceae;g__Dialister	BMI	-0.0034544	0.0450689
k__Bacteria;p__Firmicutes;c__Clostridia;o__SHA-98;f__;g__	BMI	-0.0001806	0.0106904
k__Bacteria;p__Firmicutes;c__Clostridia;o__Clostridiales;f__Lachnospiraceae;g__Anaerostipes	AB during admission	-0.0146447	0.0491344
k__Bacteria;p__Firmicutes;c__Clostridia;o__Clostridiales;f__Clostridiaceae;Other	LOS	0.0006735	0.039512
k__Bacteria;p__Firmicutes;c__Clostridia;o__Clostridiales;f__Lachnospiraceae;g__	LOS	-0.0016093	0.039512
k__Bacteria;p__Firmicutes;c__Clostridia;o__Clostridiales;f__[Tissierellaceae];g__Parvimonas	LOS	0.0002863	0.0000835
k__Bacteria;p__Proteobacteria;c__Gammaproteobacteria;o__Enterobacteriales;f__Enterobacteriaceae;g__Klebsiella	Chronic kidney failure	0.1204868	0.0000208
k__Bacteria;p__Proteobacteria;c__Gammaproteobacteria;o__Enterobacteriales;f__Enterobacteriaceae;g__Salmonella	Chronic kidney failure	0.0232286	0.0041082
k__Bacteria;p__Actinobacteria;c__Coriobacteriia;o__Coriobacteriales;f__Coriobacteriaceae;Other	Solid malignancy	0.0199917	0.0149742
k__Bacteria;p__Actinobacteria;c__Coriobacteriia;o__Coriobacteriales;f__Coriobacteriaceae;g__	Solid malignancy	0.0444445	0.0149742
k__Bacteria;p__Bacteroidetes;c__Bacteroidia;o__Bacteroidales;f__[Paraprevotellaceae];g__[Prevotella]	Solid malignancy	0.030854	0.0149742
k__Bacteria;p__Firmicutes;c__Bacilli;o__Lactobacillales;f__;g__	Solid malignancy	0.0129797	0.0302962
k__Bacteria;p__Actinobacteria;c__Coriobacteriia;o__Coriobacteriales;f__Coriobacteriaceae;g__Slackia	Immunosoppression	0.0303001	0.0482438
k__Bacteria;p__Firmicutes;c__Bacilli;o__Lactobacillales;f__;g__	Immunosoppression	0.0332543	0.0000007
k__Bacteria;p__Firmicutes;c__Bacilli;o__Lactobacillales;f__Lactobacillaceae;g__Lactobacillus	Immunosoppression	0.1074336	0.0342592
k__Bacteria;p__Firmicutes;c__Clostridia;o__Clostridiales;f__Peptococcaceae;g__Peptococcus	Immunosoppression	0.0370453	0.0328613
k__Bacteria;p__Firmicutes;c__Erysipelotrichi;o__Erysipelotrichales;f__Erysipelotrichaceae;Other	Immunosoppression	0.0286804	0.0328613
k__Bacteria;p__Firmicutes;c__Erysipelotrichi;o__Erysipelotrichales;f__Erysipelotrichaceae;g__Bulleidia	Immunosoppression	0.0407885	0.0328613
k__Bacteria;p__Firmicutes;c__Erysipelotrichi;o__Erysipelotrichales;f__Erysipelotrichaceae;g__Catenibacterium	Immunosoppression	0.2118586	0.0000001
k__Bacteria;p__Proteobacteria;c__Gammaproteobacteria;o__Enterobacteriales;f__Enterobacteriaceae;g__	Immunosoppression	0.1530103	0.0342592

Level 2

Taxon	Variable	Coefficient	P-value adjusted
k__Bacteria;Other;Other;Other;Other;Other	SDD	0.0433318	0.0004437
k__Bacteria;p__Bacteroidetes;Other;Other;Other;Other	SDD	0.0184851	0.0027675
k__Bacteria;p__Bacteroidetes;c__Bacteroidia;o__Bacteroidales;Other;Other	SDD	0.03071	0.0431657
k__Bacteria;p__Bacteroidetes;c__Bacteroidia;o__Bacteroidales;f__Prevotellaceae;Other	SDD	0.0035968	0.0431657
k__Bacteria;p__Bacteroidetes;c__Bacteroidia;o__Bacteroidales;f__Rikenellaceae;Other	SDD	0.0051633	0.0425128
k__Bacteria;p__Bacteroidetes;c__Bacteroidia;o__Bacteroidales;f__S24-7;g__	SDD	0.0276482	0.0431657
k__Bacteria;p__Bacteroidetes;c__Bacteroidia;o__Bacteroidales;f__[Paraprevotellaceae];g__	SDD	0.0096511	0.0497072
k__Bacteria;p__Firmicutes;c__Bacilli;o__Lactobacillales;f__Carnobacteriaceae;g__Granulicatella	SDD	-0.0058821	0.0425128
k__Bacteria;p__Firmicutes;c__Clostridia;Other;Other;Other	SDD	0.0087109	0.0431657
k__Bacteria;p__Firmicutes;c__Clostridia;o__Clostridiales;f__Peptococcaceae;g__Peptococcus	SDD	0.0183843	0.0497072
k__Bacteria;p__Firmicutes;c__Clostridia;o__Clostridiales;f__Ruminococcaceae;Other	SDD	-0.1082604	0.0425128
k__Bacteria;p__Firmicutes;c__Clostridia;o__Clostridiales;f__Ruminococcaceae;g__Ruminococcus	SDD	-0.0776355	0.0497072
k__Bacteria;p__Firmicutes;c__Erysipelotrichi;o__Erysipelotrichales;f__Erysipelotrichaceae;g__Bulleidia	SDD	0.0247354	0.0425128
k__Bacteria;p__Firmicutes;c__Erysipelotrichi;o__Erysipelotrichales;f__Erysipelotrichaceae;g__Catenibacterium	SDD	0.0700017	0.0377014
k__Bacteria;p__Firmicutes;c__Erysipelotrichi;o__Erysipelotrichales;f__Erysipelotrichaceae;g__Coprobacillus	SDD	-0.0267016	0.0227068
k__Bacteria;p__Proteobacteria;Other;Other;Other;Other	SDD	0.0066063	0.0425128
k__Bacteria;p__Proteobacteria;c__Betaproteobacteria;o__Burkholderiales;f__Oxalobacteraceae;g__Ralstonia	SDD	0.0053997	0.0442837
k__Bacteria;p__Proteobacteria;c__Deltaproteobacteria;o__Desulfovibrionales;f__Desulfovibrionaceae;Other	SDD	0.005289	0.0425128
k__Bacteria;p__Proteobacteria;c__Gammaproteobacteria;o__Pseudomonadales;f__Moraxellaceae;g__Acinetobacter	SDD	0.0150882	0.0460991
k__Bacteria;p__Firmicutes;c__Erysipelotrichi;o__Erysipelotrichales;f__Erysipelotrichaceae;g__	AB post admission	0.0373258	0.0316047
k__Bacteria;Other;Other;Other;Other;Other	AB during admission	0.0288833	0.0199346
k__Bacteria;p__Firmicutes;c__Clostridia;o__Clostridiales;f__Lachnospiraceae;g__Anaerostipes	AB during admission	-0.0175626	0.0199346
k__Bacteria;p__Firmicutes;c__Clostridia;o__Clostridiales;f__Ruminococcaceae;g__Ruminococcus	AB during admission	-0.0839528	0.0199346
k__Bacteria;p__Firmicutes;c__Clostridia;o__Clostridiales;f__Veillonellaceae;g__Megasphaera	AB during admission	0.0156027	0.0199346
k__Bacteria;p__Firmicutes;c__Clostridia;o__Clostridiales;f__Clostridiaceae;Other	LOS	0.0009802	0.0021279
k__Bacteria;p__Firmicutes;c__Clostridia;o__Clostridiales;f__Lachnospiraceae;g__	LOS	-0.0021579	0.0091426
k__Bacteria;p__Firmicutes;c__Clostridia;o__Clostridiales;f__Veillonellaceae;g__Veillonella	LOS	0.000529	0.0386213
k__Bacteria;p__Firmicutes;c__Clostridia;o__Clostridiales;f__[Tissierellaceae];g__Parvimonas	LOS	0.0004382	0.0000006

Supplementary material 6

	Linear component		Quadratic component	
**Taxon**	**coefficient**	**p-value**	**coefficient**	**p-value**
k__Bacteria;p__Firmicutes;c__Clostridia;o__Clostridiales;f__Lachnospiraceae;g__Roseburia	-0.010814961	0.352175714	0.131734283	1.84847E-15
k__Bacteria;p__Firmicutes;c__Erysipelotrichi;o__Erysipelotrichales;f__Erysipelotrichaceae;g__Coprobacillus	0.002927002	0.226185358	-0.021636933	5.0746E-11
k__Bacteria;p__Firmicutes;c__Bacilli;o__Lactobacillales;f__Enterococcaceae;Other	-0.000324667	0.916808115	-0.024542753	1.25275E-09
k__Bacteria;p__Firmicutes;c__Clostridia;o__Clostridiales;f__Christensenellaceae;g__	-0.000702394	0.888984222	-0.037074313	7.25994E-08
k__Bacteria;p__Firmicutes;c__Clostridia;o__Clostridiales;f__Lachnospiraceae;g__Blautia	-0.0013349	0.884036306	0.065001342	1.11686E-07
k__Bacteria;p__Firmicutes;c__Clostridia;o__Clostridiales;f__Clostridiaceae;g__Clostridium	-0.001104506	0.747814719	0.024075272	1.32535E-07
k__Bacteria;p__Firmicutes;c__Bacilli;o__Lactobacillales;f__Aerococcaceae;g__Abiotrophia	-5.08628E-05	0.901435339	-0.002684981	3.30211E-07
k__Bacteria;p__Firmicutes;c__Clostridia;o__Clostridiales;f__Christensenellaceae;g__Christensenella	2.8881E-05	0.973153215	-0.005654435	5.76676E-07
k__Bacteria;p__Firmicutes;c__Bacilli;o__Lactobacillales;f__Enterococcaceae;g__Enterococcus	-0.002010189	0.852529843	-0.068072716	7.79164E-07
k__Bacteria;p__Firmicutes;c__Bacilli;o__Lactobacillales;f__Streptococcaceae;g__Lactococcus	-0.000620758	0.664290189	-0.008825978	1.58016E-06
k__Bacteria;p__Actinobacteria;c__Coriobacteriia;o__Coriobacteriales;f__Coriobacteriaceae;g__Eggerthella	0.002319367	0.109242102	-0.009041801	2.2437E-06
k__Bacteria;p__Actinobacteria;c__Actinobacteria;o__Bifidobacteriales;f__Bifidobacteriaceae;g__Bifidobacterium	0.009624585	0.423278559	-0.0753252	2.60585E-06
k__Archaea;p__Euryarchaeota;c__Methanobacteria;o__Methanobacteriales;f__Methanobacteriaceae;g__Methanobrevibacter	-0.000970765	0.292524203	-0.005602688	8.53159E-06
k__Bacteria;p__Firmicutes;c__Clostridia;o__Clostridiales;f__Lachnospiraceae;g__	-0.018873164	0.001474051	0.033684491	1.04352E-05
k__Bacteria;p__Firmicutes;c__Clostridia;o__Clostridiales;f__Lachnospiraceae;g__Anaerostipes	-0.004990285	0.014101567	0.011070139	2.37767E-05
k__Bacteria;p__Firmicutes;c__Bacilli;o__Lactobacillales;f__Carnobacteriaceae;g__Granulicatella	0.000399485	0.542103974	-0.003586192	2.40013E-05
k__Bacteria;p__Actinobacteria;c__Actinobacteria;o__Actinomycetales;f__Micrococcaceae;g__Rothia	0.000305243	0.788739801	-0.006220601	3.47828E-05
k__Bacteria;p__Firmicutes;c__Bacilli;Other;Other;Other	0.001234206	0.428949911	-0.008187924	3.69267E-05
k__Bacteria;p__Bacteroidetes;c__Bacteroidia;o__Bacteroidales;f__[Odoribacteraceae];g__Butyricimonas	-0.000141694	0.882964888	-0.005107318	6.48255E-05
k__Bacteria;p__Firmicutes;c__Clostridia;o__Clostridiales;f__Ruminococcaceae;g__Faecalibacterium	-0.00370653	0.738314647	0.058049224	7.509E-05
k__Bacteria;p__Firmicutes;c__Clostridia;o__Clostridiales;f__Eubacteriaceae;g__Pseudoramibacter_Eubacterium	0.000845045	0.422901495	-0.00519389	0.000155269
k__Bacteria;p__Firmicutes;c__Clostridia;o__Clostridiales;f__Veillonellaceae;g__Dialister	2.85281E-07	0.9999528	0.023729933	0.00024393
k__Bacteria;p__Firmicutes;c__Clostridia;o__Clostridiales;f__Lachnospiraceae;g__Lachnospira	-0.006368446	0.123043475	0.01974404	0.000245398
k__Bacteria;p__Firmicutes;c__Bacilli;o__Lactobacillales;f__Leuconostocaceae;g__Leuconostoc	-0.00019705	0.719054032	-0.002590232	0.00030169
k__Bacteria;p__Firmicutes;c__Clostridia;o__Clostridiales;f__Lachnospiraceae;g__Dorea	-0.00884678	0.016911592	0.016868535	0.000307814
k__Bacteria;p__Firmicutes;c__Clostridia;o__Clostridiales;f__Lachnospiraceae;g__Lachnobacterium	-0.00400469	0.132965663	0.01227291	0.000374325
k__Bacteria;p__Firmicutes;c__Clostridia;o__Clostridiales;f__Lachnospiraceae;g__Coprococcus	-0.011650665	0.122598965	0.033989241	0.000427433
k__Bacteria;p__Firmicutes;c__Bacilli;o__Lactobacillales;f__Streptococcaceae;g__	0.000741446	0.568621252	-0.00568806	0.000442484
k__Bacteria;p__Verrucomicrobia;c__Verrucomicrobiae;o__Verrucomicrobiales;f__Verrucomicrobiaceae;g__Akkermansia	-0.001500496	0.688897992	-0.01664749	0.000644471
k__Bacteria;p__Bacteroidetes;c__Bacteroidia;o__Bacteroidales;f__Porphyromonadaceae;g__Parabacteroides	-0.001443458	0.761365683	-0.020863148	0.00071841
k__Bacteria;p__Firmicutes;c__Clostridia;o__Clostridiales;f__Peptococcaceae;g__Peptococcus	-0.001776645	0.269331435	-0.006947401	0.001089955
k__Bacteria;p__Firmicutes;c__Erysipelotrichi;o__Erysipelotrichales;f__Erysipelotrichaceae;Other	0.001169462	0.374103354	-0.005515548	0.001524575
k__Bacteria;p__Firmicutes;c__Clostridia;o__Clostridiales;f__[Tissierellaceae];g__Finegoldia	-0.000196396	0.706984168	-0.002072275	0.001574015
k__Bacteria;p__Firmicutes;c__Bacilli;o__Lactobacillales;Other;Other	0.003426616	0.155379439	-0.009422956	0.001802991
k__Bacteria;p__Bacteroidetes;c__Bacteroidia;o__Bacteroidales;f__[Barnesiellaceae];g__	0.000143855	0.951647994	-0.009380793	0.002656757
k__Bacteria;p__Actinobacteria;c__Coriobacteriia;o__Coriobacteriales;f__Coriobacteriaceae;g__Collinsella	-0.002280028	0.633438288	0.018732329	0.00286587
k__Bacteria;p__Bacteroidetes;c__Bacteroidia;o__Bacteroidales;f__Rikenellaceae;g__	0.004191353	0.339586339	-0.016723699	0.003329481
k__Bacteria;p__Firmicutes;c__Clostridia;o__Clostridiales;f__Ruminococcaceae;g__Anaerotruncus	0.001246739	0.097478266	-0.002682021	0.006279703
k__Bacteria;p__Firmicutes;c__Clostridia;o__Clostridiales;f__Eubacteriaceae;g__Anaerofustis	0.000473923	0.352063942	-0.001768914	0.00635898
k__Bacteria;p__Firmicutes;c__Clostridia;o__Clostridiales;f__Peptococcaceae;g__	-0.000926676	0.028480596	0.00102289	0.058167095
k__Bacteria;p__Firmicutes;c__Bacilli;o__Lactobacillales;f__Lactobacillaceae;Other	0.002161628	0.000320197	0.000645383	0.377706633
k__Bacteria;p__Tenericutes;c__Mollicutes;o__RF39;f__;g__	-0.005967977	0.000542106	0.000726466	0.737776101
